# Supplementary material for: Trace Amine-Associated Receptor 1 (TAAR1) Is a Positive Prognosticator for Epithelial Ovarian Cancer
Source: Int J Mol Sci. 2021 Aug 6;22(16):8479. doi: 10.3390/ijms22168479 (PMC8395182; doi:10.3390/ijms22168479)

Table S1. membrane TAAR1 expression correlated with selected biomarkers stained on the same patient collective

		GPCRs			Nuclear Steroid Hormone Receptors					Glycodelin (Gd)			MUC1						
		GPER	LHR	FSHR	ERα	ERβ	PRA	PRB	VDR	Gd C15	Gd Q13	GdA	115D8	HMFG1	VU3C6	VU4H5	TA-MUC1	HER2	p53
membrane TAAR1	c	-0.177	-0.071	0.019	0.342	0.172	0.256	0.250	-0.178	0.030	-0.058	-0.223	0.226	0.107	0.180	0.107	0.134	-0.052	0.168
	p	**0.042**	0.418	0.826	**0.000**	0.054	**0.004**	**0.005**	**0.046**	0.739	0.519	**0.012**	**0.013**	0.235	**0.044**	0.237	0.151	0.552	0.051
	n	132	133	129	128	126	124	126	126	126	126	126	121	125	126	125	116	135	135

Table S2. cytoplasmic TAAR1 expression correlated with selected biomarkers stained on the same patient collective

		GPCRs			Nuclear Steroid Hormone Receptors					Glycodelin (Gd)			MUC1						
		GPER	LHR	FSHR	ERα	ERβ	PRA	PRB	VDR	Gd C15	Gd Q13	GdA	115D8	HMFG1	VU3C6	VU4H5	TA-MUC1	HER2	p53
cytoplasmic TAAR1	c	0.020	-0.005	0.111	0.299	0.002	0.173	0.297	-0.045	0.116	-0.029	-0.227	-0.016	-0.045	0.029	0.059	0.119	0.040	-0.047
	p	0.823	0.950	0.210	**0.000**	0.981	**0.049**	**0.001**	0.609	0.189	0.741	**0.009**	0.855	0.610	0.744	0.506	0.192	0.647	0.592
	n	132	133	129	134	131	130	132	132	131	131	131	127	131	131	130	121	132	132

Figure S1. (a) Correlation of TAAR1 gene expression with overall survival in large independent ovarian cancer cohorts using the *KM Plotter* database ($p = 0.26$). (b) Correlation of TAAR1 gene expression with progression free survival in large independent ovarian cancer cohorts using the *KM Plotter* database ($p = 0.19$).

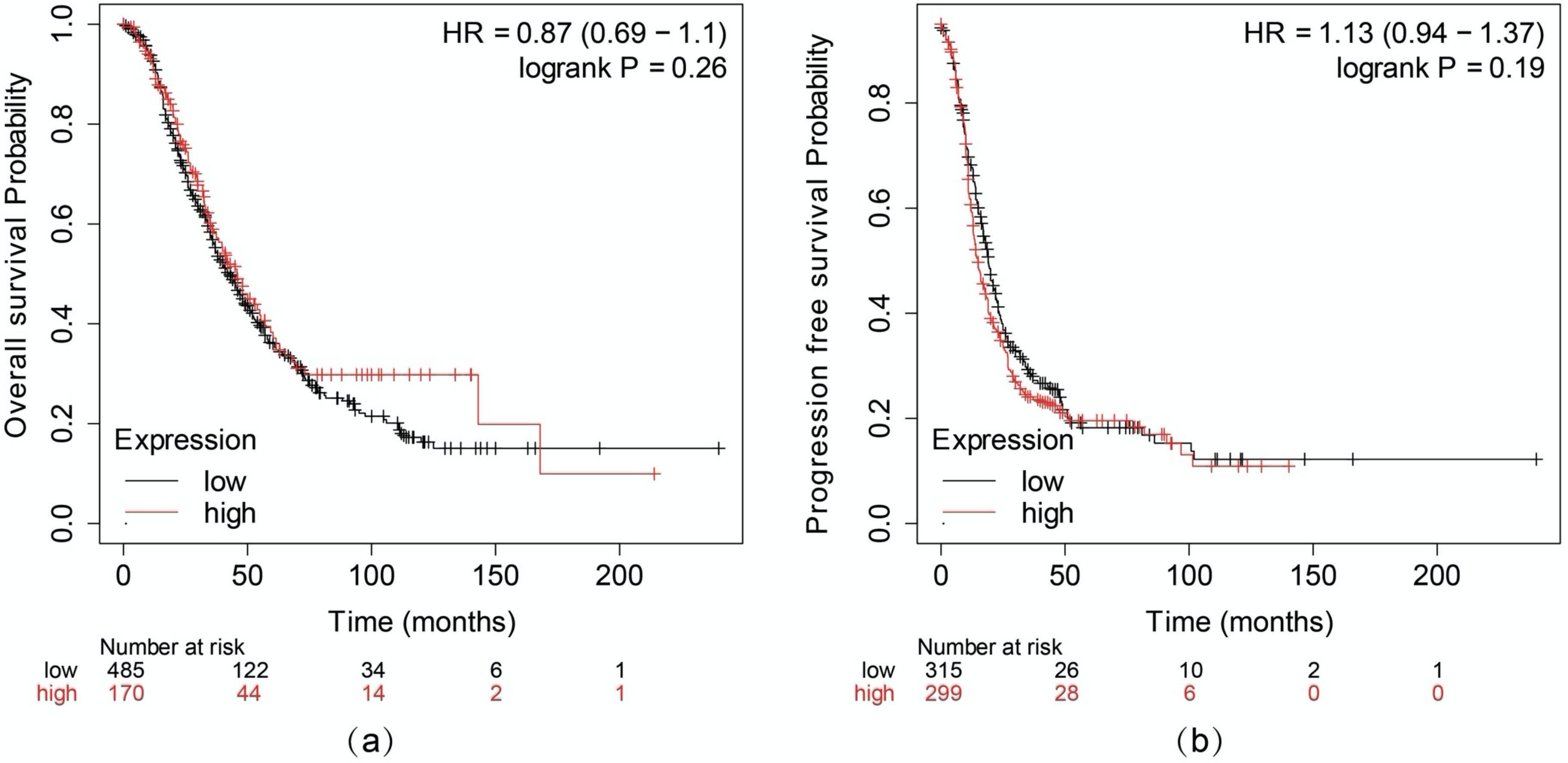

Supplement: Supplementary file 1 [file ijms-22-08479-s001.zip › ijms-1290630-supplementary.pdf]
